# Crystal structure of bromido­penta­kis­(tetra­hydro­furan-κ*O*)magnesium bis­[1,2-bis­(di­phenyl­phosphan­yl)benzene-κ^2^ *P*,*P*′]cobaltate(−1) tetra­hydro­furan disolvate

**DOI:** 10.1107/S2056989019001671

**Published:** 2019-01-31

**Authors:** Patience B. Girigiri, Stephanie H. Carpenter, William W. Brennessel, Michael L. Neidig

**Affiliations:** a Department of Chemistry, University of Rochester, Rochester, NY 14627, USA

**Keywords:** cobalt, 1,2-bis­(di­phenyl­phosphan­yl)benzene, bidentate phosphane, bis­phosphane, pseudo­tetra­hedral, backbonding, crystal structure

## Abstract

Structural characterization of the ionic title complex, [MgBr(THF)_5_][Co(dpbz)_2_]·2THF [THF is tetra­hydro­furan, C_4_H_8_O; dpbz is 1,2-bis­(di­phenyl­phosphan­yl)benzene, C_30_H_24_P_2_], revealed a well-separated cation and anion co-crystallized with two THF solvent mol­ecules that inter­act with the cation *via* weak C—H⋯O contacts. The geometry about the cobalt center is pseudo­tetra­hedral, as is expected for a *d* ^10^ metal center, only deviating from an ideal tetra­hedral geometry because of the restrictive bite angles of the bidentate phosphane ligands. Three THF ligands of the cation and one co-crystallized THF solvent mol­ecule are each disordered over two orientations. In the extended structure, the cations and THF solvent mol­ecules are arranged in (100) sheets that alternate with layers of anions, the latter of which show various π-inter­actions, which may explain the particular packing arrangement.

## Abstract 2

The reduction of CoBr_2_ by the Grignard reagent *p*-tolyl­magnesium bromide in the presence of 1,2-bis­(di­phenyl­phosphan­yl)benzene (dbpz) resulted in the *d* ^10^, formally Co^−1^ anion, [Co(dpbz)_2_]^−^. The crystal structure of the [MgBr(THF)_5_]^+^ (THF is tetra­hydro­furan) salt showed the anion to be pseudo­tetra­hedral and packed in alternating layers of anions and cations.

## Chemical context   

**Figure 1:**
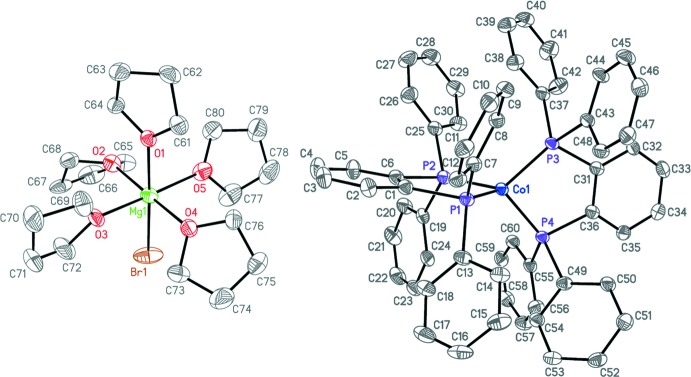
Anisotropic displacement ellipsoid plot of **1** drawn at the 50% probability level with hydrogen atoms and solvent mol­ecules omitted. Only the major component of the THF ligand disorder is shown. The reciprocal position of the two ions has been modified for clarity.

Phosphane ligands, especially aryl ones, have been used for many years to support transition metals in low oxidation states (Chatt & Watson, 1961 ▸; Chatt & Rowe, 1961 ▸). Bidentate phosphanes, or bis­phosphanes, such as 1,2-bis­(di­phenyl­phosphan­yl)benzene (dbpz), have the added benefit of the chelate effect (Cotton *et al.*, 1999 ▸). In an attempt to synthesize a cobalt(I) analog of the known iron(I) complex Fe*X*(dpbz)_2_, *X* = Cl, Br, a species proposed to be an active catalyst in Negishi cross-coupling reactions (Adams *et al.*, 2012 ▸), CoBr_2_ was reacted with four equivalents of *p*-tolylMgBr in tetra­hydro­furan (THF) at 298 K. The unexpected result was a cobalt complex in the formal −1 oxidation state, formulated as [MgBr(THF)_5_][Co(dpbz)_2_]·2THF **1** (Fig. 1 ▸). Herein we examine the crystal structure of **1** and compare it with the free bis­phosphane and related cobalt species.
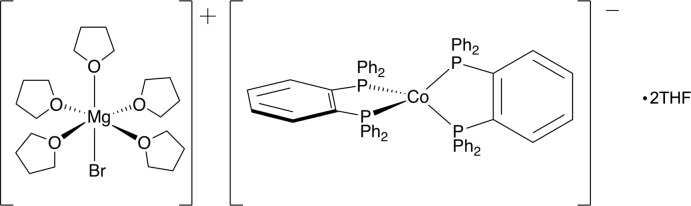


## Structural commentary   

The asymmetric unit of **1** contains one [MgBr(THF)_5_]^+^ cation, one [Co(dpbz)_2_]^−^ anion, and two co-crystallized THF solvent mol­ecules, all in general positions. The cation and anion are well separated. The average terminal P—Ph bond length in the anion of 1.859 (5) Å (Table 1 ▸) is about 0.02 Å longer than that observed in the free ligand [1.840 (2) Å, Levason *et al.*, 2006 ▸], which is consistent with backbonding from the *d* ^10^, formally Co^−1^ center into the σ* orbitals of the P—C bonds. The average terminal P—Ph bond length in **1** of 1.861 (4) Å is identical to that found in [Co(dppe)_2_]^−^ (dppe is 1,2-bis­(di­phenyl­phosphan­yl)ethane), the only other structurally characterized four-coordinate bis­(bis­phosphane) cobalt(−1) complex to date (Brennessel *et al.*, 2002 ▸).

The metal–phospho­rus bond lengths are probably the best indicator that backbonding is occurring. The average Co—P bond lengths in **1** and [Co(dppe)_2_]^−^ are 2.1014 (12) and 2.109 (1) Å, respectively. This distance increases by approximately 0.1 Å in structures containing [Co(dppe)_2_]^+^ cations, for which the cobalt center is formally in the +1 oxidation state. The average Co—P bond lengths are 2.2032 (13) and 2.1930 (6) Å, respectively, for [Co(dppe)_2_][C_60_]·1,2-di­chloro­benzene (Konarev *et al.*, 2011 ▸) and [Co(dppe)_2_][Ge_9_{Si(SiMe_3_)_3_}_3_]·C_7_H_8_ (Kysliak *et al.*, 2016 ▸). The neutral Co^0^ complex Co(dppp)_2_ (dppp = 1,2-bis­(di­phenyl­phosphan­yl)propane; Kysliak *et al.*, 2016 ▸) has an average Co—P bond length of 2.173 (1) Å, which unsurprisingly lies between that of **1** and the two Co^1+^ cations.

As is expected for a *d* ^10^ cobalt center, the geometry of **1** is essentially tetra­hedral, with a twist angle between the two P—Co—P planes of 89.465 (15) °, for which 90 ° would be ideal. The major deviation from perfect tetra­hedral geometry, however, is due to the restrictive bite angles of the dpbz ligands [average 89.97 (3)°, Table 1 ▸].

Each terminal phenyl ring from one dpbz ligand is oriented to allow for possible parallel off-center π-system inter­actions (Martinez & Iverson, 2012 ▸) with those from the second dpbz ligand. The ring pair C25–C30/C37–C42 has the shortest centroid–centroid distance of 3.5325 (16) Å and the smallest angle between ring planes of 3.26 (13)°. Ring pairs C19–C24/C55–C60 and C13–C18/C49–C54 also have reasonable distances and angles of 3.8179 (15) and 4.0796 (16) Å and 11.66 (8) and 8.67 (16)°, respectively. Only the fourth pair, C13–C18/C43–C48, seems unlikely to have any significant inter­molecular inter­action with its analogous values of 4.4142 (11) Å and 36.99 (7)°.

## Supra­molecular features   

**Figure 2:**
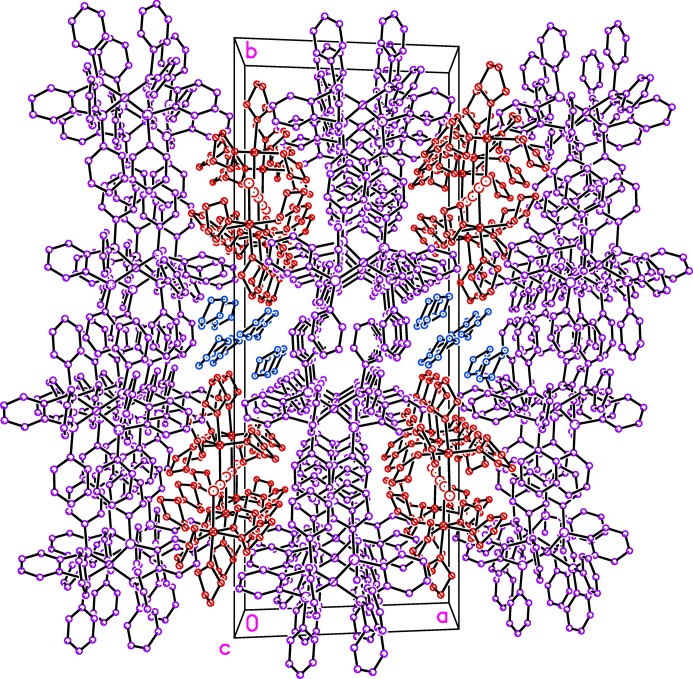
In the *bc* planes are sheets of cations (red) and THF solvent mol­ecules (blue) alternating with sheets of anions (purple). Hydrogen atoms have been omitted.

**Figure 3:**
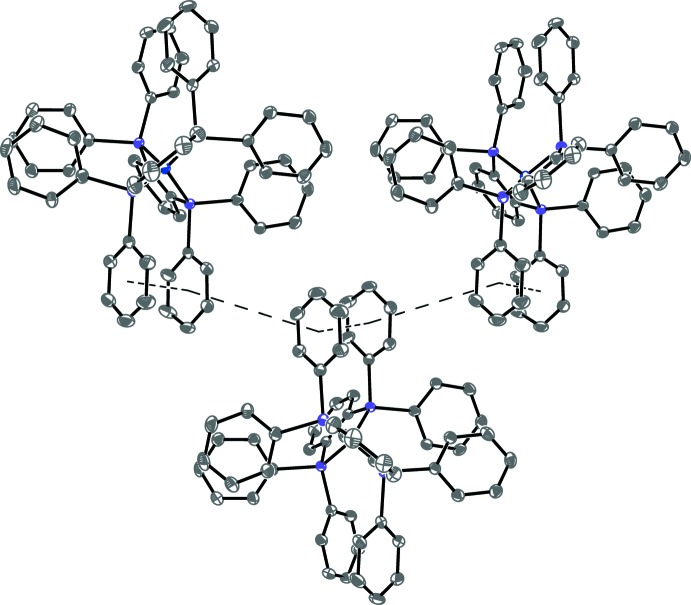
Anisotropic displacement ellipsoid plot of **1** drawn at the 50% probability level showing the extended intra- and inter­molecular π-system inter­actions with hydrogen atoms omitted. The [001] direction (*c* axis) is to the right. (See Fig. 2 ▸ for view down [001].) Symmetry-equivalent mol­ecules were generated by crystallographic twofold screw axes with symmetry operators *x*, 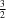 − *y*, −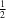 + *z* and *x*, 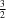 − *y*, 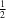 + *z*.

**Figure 4:**
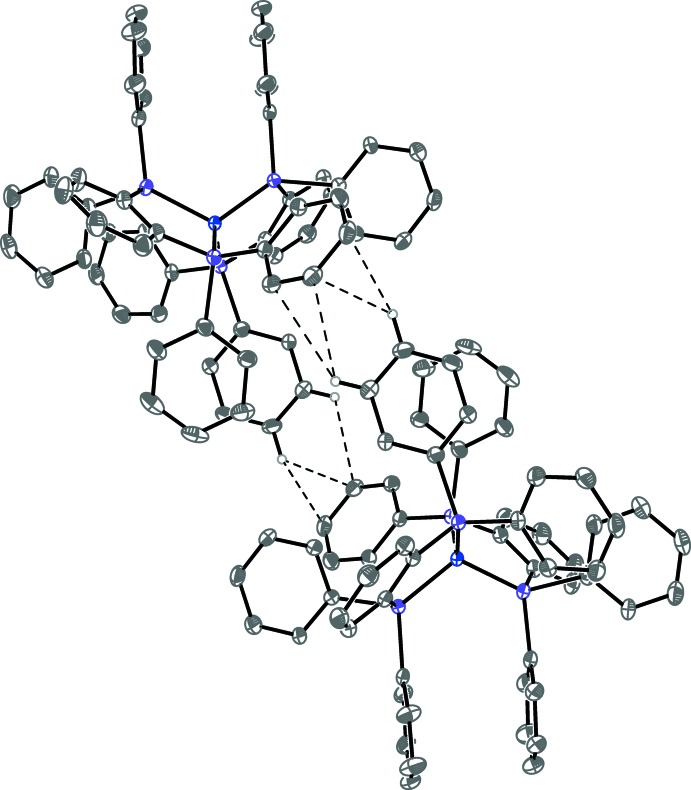
Anisotropic displacement ellipsoid plot of **1** drawn at the 50% probability level of the edge-to-face π-system contacts that link the chains aligned along [001] in the [010] direction as well, thus offering an explanation for the observed two-dimensional sheets of anions. Hydrogen atoms except for those on carbon atoms C51 and C52 (and their symmetry equivalents) were omitted. The symmetry-equivalent mol­ecule was generated by a crystallographic inversion center with symmetry operator 1 − *x*, 2 − *y*, 1 − *z*.

Both co-crystallized THF solvent mol­ecules inter­act with the cation *via* weak C—H⋯O bonds (Table 2 ▸). The cations and THF mol­ecules of solvation are found in sheets normal to [100] that alternate with sheets of the anions (Fig. 2 ▸). Within each layer of anions there appear to be numerous potential π-system inter­actions (Martinez & Iverson, 2012 ▸; McGaughey *et al.*, 1998 ▸). Along [001] is an alternation between short intra­molecular offset parallel stacking and longer inter­molecular inter­actions with centroid–centroid distances of 3.533 (2) and 5.252 (2) Å, respectively (Fig. 3 ▸). On the opposite side of each mol­ecule and also along the [001] direction is a second analogous set of potential π-system inter­actions, but with longer centroid–centroid distances of 4.080 (2) and 5.786 (2) Å; however, these rings are nearly coplanar (*i.e*. the open faces are not directed toward one another) and therefore they are unlikely to have any significant attractive inter­molecular inter­actions. Upon further inspection, the one-dimensional chains along [001] are linked to other parallel chains by phenyl rings that are oriented correctly for edge-to-face C—H⋯π attractive inter­actions (Fig. 4 ▸), thus providing a possible explanation for the two-dimensional packing motif of anions in the *bc* planes.

## Database survey   

The only other structure containing a four-coordinate cobalt(−1) anion with two aryl bis­phosphanes is the potassium 18-crown-6 salt of [Co(dppe)_2_]^−^ (Brennessel *et al.*, 2002 ▸). Multiple species containing four-coordinate metals with two dpbz ligands are found in the Cambridge Structural Database (CSD, Version 5.40, November 2018; Groom *et al.*, 2016 ▸) with the following counts: Ni(dpbz)_2_: five, Pt(dpbz)_2_: two, [Cu(dpbz)_2_]^+^: one, [Ag(dpbz)_2_]^+^: five, [Au(dpbz)_2_]^+^: thirteen. Additionally there is one occurrence each of the square-planar cations [Rh(dpbz)_2_]^+^ and [Ni(dpbz)_2_]^2+^.

## Synthesis and crystallization   

CoBr_2_ (99%, Sigma–Aldrich), dpbz (98%, Strem), *p*-tolyl­MgBr (1.0 *M* in THF, Sigma–Aldrich), THF (Sigma–Aldrich, anhydrous, 99.9%, inhibitor-free), and *n*-pentane (Sigma–Aldrich, >99%, anhydrous) were used in the synthesis of **1** without further purification. All reactions were performed in an MBraun inert-atmosphere (N_2_) glovebox. CoBr_2_ (27 mg, 0.12 mmol) and dpbz (99 mg, 0.22 mmol, 1.8 equiv.) were dissolved in 1 mL THF. *p*-TolylMgBr (494 µL, 4 equiv.) was added to the cobalt solution at 0.33 mmol min^−1^ at room temperature. The resulting dark-red solution was allowed to stir at room temperature at 770 r.p.m. for 30 min. The solution was then filtered through Celite. Pentane (1 mL) was layered on top of the solution, and the solution was stored in a 243 K freezer until orange–brown crystalline blocks of **1** were observed.

## Refinement   

Crystal data, data collection and structure refinement details are summarized in Table 3 ▸. Three THF ligands and one co-crystallized THF solvent mol­ecule were modeled as disordered over two sets of site each: O2/C65–C68, 0.650 (8):0.350 (8), O3/C69–C72, 0.615 (8):0.385 (8), O5/C77–C80, 0.63 (2):0.37 (2), O7/C85–C88, 0.609 (4):0.391 (4). Analogous bond lengths and angles between the two positions of each disordered THF mol­ecule were restrained to be similar. Anisotropic displacement parameters for proximal atoms were constrained to be equivalent.

H atoms were refined using riding models: aromatic, C—H = 0.93 Å, and methyl­ene, C—H = 0.97 Å, with *U*_iso_(H) = 1.2*U*_eq_(C).

The maximum residual peak of 0.64 e Å^−3^ and the deepest hole of −1.69 e Å^−3^ are found 0.84 and 0.83 Å from atoms H74*A* and Br1, respectively.

## Supplementary Material

Crystal structure: contains datablock(s) I, global. DOI: 10.1107/S2056989019001671/hb7799sup1.cif

Structure factors: contains datablock(s) I. DOI: 10.1107/S2056989019001671/hb7799Isup2.hkl

CCDC reference: 1894405

Additional supporting information:  crystallographic information; 3D view; checkCIF report

## Figures and Tables

**Table 1:** Selected geometric parameters (Å, °)

Co1—P1	2.1049 (6)	P2—C19	1.867 (2)
Co1—P2	2.0988 (6)	P2—C25	1.847 (2)
Co1—P3	2.0968 (6)	P3—C31	1.859 (2)
Co1—P4	2.1050 (6)	P3—C37	1.846 (2)
P1—C1	1.870 (2)	P3—C43	1.870 (2)
P1—C7	1.876 (2)	P4—C36	1.863 (2)
P1—C13	1.849 (2)	P4—C49	1.843 (2)
P2—C6	1.854 (2)	P4—C55	1.874 (2)
			
P1—Co1—P4	120.60 (3)	P3—Co1—P1	122.08 (3)
P2—Co1—P1	90.18 (2)	P3—Co1—P2	118.74 (3)
P2—Co1—P4	118.59 (2)	P3—Co1—P4	89.75 (2)

**Table 2:** Hydrogen-bond geometry (Å, °)

*D*—H⋯*A*	*D*—H	H⋯*A*	*D*⋯*A*	*D*—H⋯*A*
C62—H62*B*⋯O6	0.97	2.48	3.438 (4)	167
C63—H63*B*⋯O7	0.97	2.59	3.555 (8)	179
C63—H63*B*⋯O7′	0.97	2.63	3.565 (6)	162

**Table 3:** Experimental details

Crystal data	
Chemical formula	[MgBr(C_4_H_8_O)_5_][Co(C_30_H_24_P_2_)_2_]·2C_4_H_8_O
*M*_r_	1560.74
Crystal system, space group	Monoclinic, *P*2_1_/*c*
Temperature (K)	100
*a*, *b*, *c* (Å)	15.1096 (2), 38.1917 (3), 14.1266 (1)
β (°)	106.102 (1)
*V* (Å^3^)	7832.11 (14)
*Z*	4
Radiation type	Cu *K*α
μ (mm^−1^)	3.60
Crystal size (mm)	0.42 × 0.13 × 0.07

Data collection	
Diffractometer	Rigaku XtaLAB Synergy, Dualflex, HyPix
Absorption correction	Multi-scan (*CrysAlis PRO*; Rigaku OD, 2018 ▸)
*T*_min_, *T*_max_	0.290, 1.000
No. of measured, independent and observed [*I* > 2σ(*I*)] reflections	73364, 16393, 14853
*R*_int_	0.048
(sin θ/λ)_max_ (Å^−1^)	0.634

Refinement	
*R*[*F* ^2^ > 2σ(*F* ^2^)], *wR*(*F* ^2^), *S*	0.048, 0.127, 1.06
No. of reflections	16393
No. of parameters	974
No. of restraints	91
H-atom treatment	H-atom parameters constrained
Δρ_max_, Δρ_min_ (e Å^−3^)	0.64, −1.69

Computer programs: *CrysAlis PRO* (Rigaku OD, 2018 ▸), *SHELXT* (Sheldrick, 2015*a* ▸), *SHELXL* (Sheldrick, 2015*b* ▸) and *OLEX2* (Dolomanov *et al.*, 2009 ▸).

## supplementary crystallographic information

### Crystal data

**Table:**

[MgBr(C_4_H_8_O)_5_][Co(C_30_H_24_P_2_)_2_]·2C_4_H_8_O	*F*(000) = 3288
*M**_r_* = 1560.74	*D*_x_ = 1.324 Mg m^−^^3^
Monoclinic, *P*2_1_/*c*	Cu *K*α radiation, λ = 1.54184 Å
*a* = 15.1096 (2) Å	Cell parameters from 33363 reflections
*b* = 38.1917 (3) Å	θ = 3.5–77.4°
*c* = 14.1266 (1) Å	µ = 3.60 mm^−^^1^
β = 106.102 (1)°	*T* = 100 K
*V* = 7832.11 (14) Å^3^	Block, brown
*Z* = 4	0.42 × 0.13 × 0.07 mm

### Data collection

**Table:**

Rigaku XtaLAB Synergy, Dualflex, HyPix diffractometer	16393 independent reflections
Radiation source: micro-focus sealed X-ray tube, PhotonJet (Cu) X-ray Source	14853 reflections with *I* > 2σ(*I*)
Mirror monochromator	*R*_int_ = 0.048
ω scans	θ_max_ = 77.9°, θ_min_ = 2.3°
Absorption correction: multi-scan (CrysAlisPro; Rigaku OD, 2018)	*h* = −17→19
*T*_min_ = 0.290, *T*_max_ = 1.000	*k* = −48→42
73364 measured reflections	*l* = −17→17

### Refinement

**Table:**

Refinement on *F*^2^	Primary atom site location: dual
Least-squares matrix: full	Secondary atom site location: difference Fourier map
*R*[*F*^2^ > 2σ(*F*^2^)] = 0.048	Hydrogen site location: inferred from neighbouring sites
*wR*(*F*^2^) = 0.127	H-atom parameters constrained
*S* = 1.06	*w* = 1/[σ^2^(*F*_o_^2^) + (0.0611*P*)^2^ + 8.1124*P*] where *P* = (*F*_o_^2^ + 2*F*_c_^2^)/3
16393 reflections	(Δ/σ)_max_ = 0.002
974 parameters	Δρ_max_ = 0.64 e Å^−^^3^
91 restraints	Δρ_min_ = −1.69 e Å^−^^3^

### Special details

**Table:**

Geometry. All esds (except the esd in the dihedral angle between two l.s. planes) are estimated using the full covariance matrix. The cell esds are taken into account individually in the estimation of esds in distances, angles and torsion angles; correlations between esds in cell parameters are only used when they are defined by crystal symmetry. An approximate (isotropic) treatment of cell esds is used for estimating esds involving l.s. planes.
Refinement. Three THF ligands and one cocrystallized THF solvent molecule are modeled as disordered over two positions: O2/C65-C68, 0.650 (8):0.350 (8), O3/C69-C72, 0.615 (8):0.385 (8), O5/C77-C80, 0.63 (2):0.37 (2), O7/C85-C88, 0.609 (4):0.391 (4). Analogous bond lengths and angles between the two positions of each disordered THF molecule were restrained to be similar. Anisotropic displacement parameters for proximal atoms were constrained to be equivalent.

### Fractional atomic coordinates and isotropic or equivalent isotropic displacement parameters (Å^2^)

**Table:**

	*x*	*y*	*z*	*U*_iso_*/*U*_eq_	Occ. (<1)
Co1	0.56200 (2)	0.88268 (2)	0.29552 (2)	0.01606 (9)	
P1	0.67543 (4)	0.90934 (2)	0.38763 (4)	0.01799 (11)	
P2	0.64893 (4)	0.85876 (2)	0.22175 (4)	0.01868 (11)	
P3	0.47536 (4)	0.85100 (2)	0.35207 (4)	0.01739 (11)	
P4	0.44633 (4)	0.91051 (2)	0.21208 (4)	0.01730 (11)	
C1	0.78342 (16)	0.89463 (6)	0.35948 (16)	0.0219 (4)	
C2	0.87300 (17)	0.90505 (7)	0.40934 (18)	0.0271 (5)	
H2	0.882334	0.920804	0.461381	0.032*	
C3	0.94796 (18)	0.89211 (7)	0.38189 (19)	0.0321 (5)	
H3	1.007331	0.898804	0.416351	0.038*	
C4	0.93442 (19)	0.86910 (8)	0.3028 (2)	0.0352 (6)	
H4	0.984716	0.860446	0.284349	0.042*	
C5	0.84630 (17)	0.85912 (7)	0.25175 (18)	0.0284 (5)	
H5	0.837316	0.844301	0.197676	0.034*	
C6	0.77029 (16)	0.87121 (6)	0.28094 (16)	0.0228 (4)	
C7	0.71708 (15)	0.90607 (6)	0.52539 (15)	0.0200 (4)	
C8	0.68730 (16)	0.87745 (6)	0.56995 (16)	0.0216 (4)	
H8	0.649168	0.860760	0.531045	0.026*	
C9	0.71407 (17)	0.87364 (6)	0.67193 (17)	0.0262 (5)	
H9	0.692940	0.854599	0.700506	0.031*	
C10	0.77197 (17)	0.89796 (7)	0.73140 (17)	0.0264 (5)	
H10	0.789580	0.895353	0.799533	0.032*	
C11	0.80335 (17)	0.92628 (6)	0.68783 (17)	0.0248 (5)	
H11	0.843014	0.942527	0.726858	0.030*	
C12	0.77547 (16)	0.93034 (6)	0.58588 (16)	0.0227 (4)	
H12	0.796104	0.949558	0.557585	0.027*	
C13	0.68770 (16)	0.95732 (6)	0.37907 (15)	0.0223 (4)	
C14	0.64101 (17)	0.97900 (6)	0.42872 (16)	0.0246 (5)	
H14	0.604953	0.969028	0.465297	0.029*	
C15	0.64753 (19)	1.01517 (6)	0.42440 (18)	0.0307 (5)	
H15	0.617267	1.029186	0.459358	0.037*	
C16	0.6992 (2)	1.03052 (6)	0.36797 (19)	0.0343 (6)	
H16	0.704354	1.054750	0.365632	0.041*	
C17	0.74250 (19)	1.00939 (7)	0.31555 (19)	0.0346 (6)	
H17	0.775663	1.019538	0.276206	0.042*	
C18	0.73743 (18)	0.97309 (6)	0.32057 (17)	0.0277 (5)	
H18	0.767287	0.959235	0.284812	0.033*	
C19	0.64638 (16)	0.87156 (6)	0.09346 (16)	0.0227 (4)	
C20	0.65586 (18)	0.84885 (7)	0.01949 (17)	0.0280 (5)	
H20	0.667014	0.825189	0.033357	0.034*	
C21	0.6487 (2)	0.86137 (8)	−0.07511 (19)	0.0346 (6)	
H21	0.653822	0.845911	−0.124195	0.042*	
C22	0.63415 (19)	0.89669 (8)	−0.09651 (18)	0.0330 (6)	
H22	0.629573	0.904947	−0.159615	0.040*	
C23	0.62645 (17)	0.91957 (7)	−0.02309 (18)	0.0291 (5)	
H23	0.617585	0.943347	−0.036667	0.035*	
C24	0.63193 (16)	0.90709 (6)	0.07081 (17)	0.0240 (5)	
H24	0.625892	0.922629	0.119317	0.029*	
C25	0.65782 (16)	0.81068 (6)	0.21354 (16)	0.0224 (4)	
C26	0.73116 (18)	0.79079 (7)	0.26938 (19)	0.0304 (5)	
H26	0.782408	0.801993	0.309945	0.036*	
C27	0.7287 (2)	0.75444 (7)	0.2652 (2)	0.0388 (6)	
H27	0.778131	0.741617	0.303426	0.047*	
C28	0.6540 (2)	0.73719 (7)	0.2053 (2)	0.0368 (6)	
H28	0.653172	0.712870	0.201900	0.044*	
C29	0.5805 (2)	0.75637 (7)	0.15036 (19)	0.0319 (5)	
H29	0.529601	0.744936	0.109823	0.038*	
C30	0.58174 (18)	0.79270 (6)	0.15510 (17)	0.0274 (5)	
H30	0.530978	0.805271	0.118672	0.033*	
C31	0.35262 (16)	0.86047 (6)	0.28835 (15)	0.0201 (4)	
C32	0.27655 (16)	0.84320 (6)	0.30413 (17)	0.0231 (4)	
H32	0.285582	0.824841	0.349060	0.028*	
C33	0.18766 (17)	0.85306 (6)	0.25364 (18)	0.0272 (5)	
H33	0.137412	0.840906	0.262961	0.033*	
C34	0.17416 (17)	0.88139 (6)	0.18868 (18)	0.0266 (5)	
H34	0.114784	0.888345	0.155026	0.032*	
C35	0.24949 (16)	0.89911 (6)	0.17452 (16)	0.0229 (4)	
H35	0.240158	0.918037	0.131435	0.028*	
C36	0.33930 (16)	0.88898 (6)	0.22401 (15)	0.0200 (4)	
C37	0.48157 (16)	0.80276 (6)	0.34774 (15)	0.0202 (4)	
C38	0.56174 (17)	0.78692 (6)	0.40489 (17)	0.0256 (5)	
H38	0.609041	0.800857	0.442388	0.031*	
C39	0.57257 (19)	0.75087 (6)	0.40711 (17)	0.0290 (5)	
H39	0.625634	0.740837	0.447858	0.035*	
C40	0.5045 (2)	0.72977 (6)	0.34878 (19)	0.0313 (5)	
H40	0.511021	0.705546	0.350722	0.038*	
C41	0.4267 (2)	0.74522 (7)	0.2875 (2)	0.0373 (6)	
H41	0.381890	0.731312	0.245942	0.045*	
C42	0.41491 (19)	0.78124 (6)	0.28750 (19)	0.0308 (5)	
H42	0.361715	0.791149	0.246673	0.037*	
C43	0.46386 (16)	0.85598 (5)	0.47980 (16)	0.0199 (4)	
C44	0.45917 (17)	0.82847 (6)	0.54279 (16)	0.0237 (5)	
H44	0.455585	0.805586	0.519505	0.028*	
C45	0.45976 (19)	0.83461 (6)	0.64000 (18)	0.0294 (5)	
H45	0.459109	0.815859	0.681794	0.035*	
C46	0.46132 (18)	0.86856 (7)	0.67472 (17)	0.0270 (5)	
H46	0.462255	0.872724	0.739850	0.032*	
C47	0.46148 (17)	0.89631 (6)	0.61135 (17)	0.0249 (5)	
H47	0.460033	0.919183	0.633482	0.030*	
C48	0.46377 (16)	0.89022 (6)	0.51557 (16)	0.0222 (4)	
H48	0.465280	0.909073	0.474367	0.027*	
C49	0.42490 (16)	0.95620 (6)	0.24138 (15)	0.0207 (4)	
C50	0.36400 (16)	0.96529 (6)	0.29604 (16)	0.0228 (4)	
H50	0.331658	0.947800	0.317968	0.027*	
C51	0.35115 (18)	0.99999 (6)	0.31800 (18)	0.0278 (5)	
H51	0.310294	1.005477	0.354377	0.033*	
C52	0.39851 (18)	1.02656 (6)	0.28634 (18)	0.0269 (5)	
H52	0.388231	1.049855	0.299325	0.032*	
C53	0.46147 (17)	1.01792 (6)	0.23501 (17)	0.0254 (5)	
H53	0.494433	1.035528	0.214478	0.030*	
C54	0.47575 (16)	0.98322 (6)	0.21399 (16)	0.0219 (4)	
H54	0.519613	0.977807	0.181308	0.026*	
C55	0.41173 (15)	0.91347 (6)	0.07424 (15)	0.0207 (4)	
C56	0.38055 (17)	0.94338 (6)	0.01859 (16)	0.0253 (5)	
H56	0.371206	0.963866	0.050098	0.030*	
C57	0.36315 (18)	0.94309 (7)	−0.08338 (17)	0.0287 (5)	
H57	0.343390	0.963431	−0.119193	0.034*	
C58	0.37506 (18)	0.91275 (7)	−0.13187 (16)	0.0282 (5)	
H58	0.363781	0.912604	−0.200015	0.034*	
C59	0.40407 (17)	0.88249 (6)	−0.07749 (17)	0.0254 (5)	
H59	0.411482	0.861881	−0.109503	0.030*	
C60	0.42199 (16)	0.88287 (6)	0.02395 (16)	0.0218 (4)	
H60	0.441202	0.862402	0.059370	0.026*	
Br1	0.07583 (2)	0.75114 (2)	0.27391 (3)	0.05868 (12)	
Mg1	0.07915 (6)	0.68385 (2)	0.30221 (6)	0.02865 (18)	
O1	0.09259 (12)	0.62946 (4)	0.33147 (12)	0.0261 (3)	
O2	0.2044 (6)	0.6882 (4)	0.4168 (6)	0.0328 (14)	0.650 (8)
C65	0.2808 (13)	0.7075 (4)	0.3963 (9)	0.0435 (19)	0.650 (8)
H65A	0.260351	0.719528	0.333538	0.052*	0.650 (8)
H65B	0.330335	0.691638	0.394153	0.052*	0.650 (8)
C66	0.3132 (7)	0.7336 (2)	0.4795 (7)	0.061 (2)	0.650 (8)
H66A	0.312449	0.757270	0.454073	0.073*	0.650 (8)
H66B	0.375151	0.728174	0.519051	0.073*	0.650 (8)
C67	0.2439 (5)	0.72976 (13)	0.5401 (4)	0.0528 (15)	0.650 (8)
H67A	0.272513	0.734185	0.609470	0.063*	0.650 (8)
H67B	0.191779	0.745358	0.516600	0.063*	0.650 (8)
C68	0.2156 (6)	0.69171 (19)	0.5216 (6)	0.0409 (17)	0.650 (8)
H68A	0.263053	0.676108	0.559338	0.049*	0.650 (8)
H68B	0.158368	0.687026	0.537742	0.049*	0.650 (8)
O2'	0.2129 (11)	0.6891 (7)	0.4020 (12)	0.0328 (14)	0.350 (8)
C65'	0.283 (3)	0.7096 (8)	0.3735 (18)	0.0435 (19)	0.350 (8)
H65C	0.259385	0.718214	0.306607	0.052*	0.350 (8)
H65D	0.337444	0.695667	0.378115	0.052*	0.350 (8)
C66'	0.3036 (16)	0.7397 (5)	0.4469 (13)	0.061 (2)	0.350 (8)
H66C	0.366119	0.748169	0.457361	0.073*	0.350 (8)
H66D	0.260966	0.759007	0.425123	0.073*	0.350 (8)
C67'	0.2905 (10)	0.7227 (3)	0.5392 (8)	0.0528 (15)	0.350 (8)
H67C	0.349808	0.718043	0.585804	0.063*	0.350 (8)
H67D	0.255683	0.738016	0.570256	0.063*	0.350 (8)
C68'	0.2388 (12)	0.6888 (4)	0.5082 (12)	0.0409 (17)	0.350 (8)
H68C	0.277851	0.668846	0.533571	0.049*	0.350 (8)
H68D	0.184670	0.687730	0.532226	0.049*	0.350 (8)
O3	−0.0021 (10)	0.6865 (6)	0.4036 (10)	0.034 (2)	0.615 (8)
C69	−0.053 (2)	0.6572 (5)	0.429 (2)	0.0478 (18)	0.615 (8)
H69A	−0.015193	0.636296	0.440744	0.057*	0.615 (8)
H69B	−0.108062	0.652537	0.375647	0.057*	0.615 (8)
C70	−0.0782 (5)	0.6678 (2)	0.5203 (5)	0.0741 (19)	0.615 (8)
H70A	−0.134850	0.656603	0.523682	0.089*	0.615 (8)
H70B	−0.029452	0.661916	0.579042	0.089*	0.615 (8)
C71	−0.0899 (8)	0.7065 (3)	0.5088 (7)	0.092 (3)	0.615 (8)
H71A	−0.150997	0.712406	0.467801	0.111*	0.615 (8)
H71B	−0.079965	0.717898	0.572281	0.111*	0.615 (8)
C72	−0.018 (3)	0.7169 (6)	0.460 (3)	0.069 (3)	0.615 (8)
H72A	−0.038528	0.736769	0.417164	0.083*	0.615 (8)
H72B	0.038596	0.723300	0.509514	0.083*	0.615 (8)
O3'	0.0124 (19)	0.6851 (10)	0.4153 (18)	0.034 (2)	0.385 (8)
C69'	−0.054 (3)	0.6578 (9)	0.420 (4)	0.0478 (18)	0.385 (8)
H69C	−0.023401	0.637953	0.457594	0.057*	0.385 (8)
H69D	−0.086481	0.649934	0.353927	0.057*	0.385 (8)
C70'	−0.1195 (8)	0.6747 (4)	0.4689 (8)	0.0741 (19)	0.385 (8)
H70C	−0.170933	0.685534	0.421139	0.089*	0.385 (8)
H70D	−0.142676	0.657841	0.507567	0.089*	0.385 (8)
C71'	−0.0591 (12)	0.7015 (5)	0.5333 (13)	0.092 (3)	0.385 (8)
H71C	−0.016780	0.690800	0.590404	0.111*	0.385 (8)
H71D	−0.095267	0.719204	0.554886	0.111*	0.385 (8)
C72'	−0.009 (5)	0.7164 (9)	0.464 (5)	0.069 (3)	0.385 (8)
H72C	−0.048369	0.732368	0.417296	0.083*	0.385 (8)
H72D	0.046244	0.728652	0.499914	0.083*	0.385 (8)
O4	−0.04687 (12)	0.67674 (5)	0.19181 (13)	0.0308 (4)	
O5	0.14741 (14)	0.67323 (6)	0.19244 (15)	0.0393 (4)	
C61	0.0442 (2)	0.60163 (7)	0.2688 (2)	0.0357 (6)	
H61A	0.048397	0.604591	0.201990	0.043*	
H61B	−0.020300	0.601421	0.267637	0.043*	
C62	0.0908 (2)	0.56810 (7)	0.3130 (2)	0.0388 (6)	
H62A	0.143673	0.562937	0.288974	0.047*	
H62B	0.048477	0.548477	0.298570	0.047*	
C63	0.1199 (2)	0.57638 (7)	0.4218 (2)	0.0378 (6)	
H63A	0.170251	0.561467	0.457036	0.045*	
H63B	0.068850	0.573869	0.450490	0.045*	
C64	0.15005 (19)	0.61415 (6)	0.42258 (19)	0.0307 (5)	
H64A	0.140981	0.626320	0.479427	0.037*	
H64B	0.214657	0.615542	0.424626	0.037*	
C73	−0.1252 (2)	0.69921 (7)	0.1900 (2)	0.0387 (6)	
H73A	−0.104208	0.721455	0.221390	0.046*	
H73B	−0.164713	0.688165	0.224965	0.046*	
C74	−0.1766 (3)	0.70470 (10)	0.0843 (3)	0.0631 (10)	
H74A	−0.174948	0.729176	0.066577	0.076*	
H74B	−0.240390	0.697599	0.072408	0.076*	
C75	−0.1287 (2)	0.68231 (8)	0.0248 (2)	0.0430 (7)	
H75A	−0.089869	0.696463	−0.004307	0.052*	
H75B	−0.173152	0.669851	−0.027032	0.052*	
C76	−0.0716 (2)	0.65707 (7)	0.10046 (19)	0.0340 (6)	
H76A	−0.107210	0.636466	0.106415	0.041*	
H76B	−0.016999	0.649800	0.082313	0.041*	
C77	0.1439 (18)	0.6960 (4)	0.1093 (10)	0.0552 (19)	0.63 (2)
H77A	0.191600	0.713717	0.126867	0.066*	0.63 (2)
H77B	0.084462	0.707410	0.087106	0.066*	0.63 (2)
C78	0.1598 (10)	0.6716 (3)	0.0293 (8)	0.065 (2)	0.63 (2)
H78A	0.102009	0.665872	−0.018558	0.077*	0.63 (2)
H78B	0.200498	0.682462	−0.004627	0.077*	0.63 (2)
C77'	0.136 (3)	0.6945 (7)	0.1048 (19)	0.0552 (19)	0.37 (2)
H77C	0.156540	0.718280	0.122321	0.066*	0.37 (2)
H77D	0.071724	0.695078	0.066605	0.066*	0.37 (2)
C78'	0.1951 (15)	0.6769 (3)	0.0463 (15)	0.065 (2)	0.37 (2)
H78C	0.254901	0.688101	0.058996	0.077*	0.37 (2)
H78D	0.164921	0.677615	−0.023935	0.077*	0.37 (2)
C79	0.2041 (2)	0.63911 (11)	0.0853 (2)	0.0565 (9)	
H79A	0.163914	0.618969	0.066437	0.068*	0.63 (2)
H79B	0.262407	0.634057	0.072041	0.068*	0.63 (2)
H79C	0.148479	0.625563	0.058270	0.068*	0.37 (2)
H79D	0.256559	0.627066	0.073572	0.068*	0.37 (2)
C80	0.2183 (2)	0.64766 (9)	0.1924 (2)	0.0442 (7)	
H80A	0.211180	0.626936	0.229260	0.053*	
H80B	0.279190	0.657418	0.220931	0.053*	
O6	−0.05333 (17)	0.49866 (6)	0.22536 (16)	0.0474 (5)	
C81	−0.0188 (3)	0.49417 (10)	0.1421 (2)	0.0534 (8)	
H81A	0.038407	0.481068	0.160298	0.064*	
H81B	−0.007326	0.516744	0.116385	0.064*	
C82	−0.0908 (2)	0.47449 (9)	0.0663 (3)	0.0492 (8)	
H82A	−0.062744	0.458644	0.029350	0.059*	
H82B	−0.131323	0.490446	0.020755	0.059*	
C83	−0.1428 (3)	0.45449 (9)	0.1261 (3)	0.0595 (10)	
H83A	−0.208385	0.459082	0.102317	0.071*	
H83B	−0.132509	0.429510	0.122733	0.071*	
C84	−0.1046 (2)	0.46776 (10)	0.2301 (3)	0.0582 (9)	
H84A	−0.154348	0.472953	0.259008	0.070*	
H84B	−0.065064	0.450215	0.270418	0.070*	
O7	−0.0684 (6)	0.5669 (2)	0.5248 (6)	0.0581 (11)	0.391 (4)
C85	−0.1238 (7)	0.5650 (3)	0.5912 (8)	0.0498 (14)	0.391 (4)
H85A	−0.171262	0.582890	0.575925	0.060*	0.391 (4)
H85B	−0.086465	0.568282	0.658530	0.060*	0.391 (4)
C86	−0.1665 (8)	0.5290 (3)	0.5781 (8)	0.059 (2)	0.391 (4)
H86A	−0.127405	0.512239	0.622100	0.071*	0.391 (4)
H86B	−0.226620	0.529216	0.590166	0.071*	0.391 (4)
C87	−0.1741 (10)	0.5205 (3)	0.4720 (9)	0.085 (2)	0.391 (4)
H87A	−0.170803	0.495447	0.461927	0.102*	0.391 (4)
H87B	−0.230730	0.529562	0.428331	0.102*	0.391 (4)
C88	−0.0918 (8)	0.5389 (3)	0.4575 (8)	0.0597 (19)	0.391 (4)
H88A	−0.040437	0.522732	0.468120	0.072*	0.391 (4)
H88B	−0.105216	0.547718	0.390648	0.072*	0.391 (4)
O7'	−0.0982 (4)	0.57383 (12)	0.4729 (4)	0.0581 (11)	0.609 (4)
C85'	−0.1206 (4)	0.58116 (18)	0.5618 (4)	0.0498 (14)	0.609 (4)
H85C	−0.173875	0.596458	0.549324	0.060*	0.609 (4)
H85D	−0.069352	0.592606	0.608368	0.060*	0.609 (4)
C86'	−0.1416 (5)	0.5462 (2)	0.6032 (5)	0.059 (2)	0.609 (4)
H86C	−0.100306	0.542011	0.668204	0.071*	0.609 (4)
H86D	−0.204677	0.545470	0.607165	0.071*	0.609 (4)
C87'	−0.1266 (8)	0.51961 (19)	0.5302 (6)	0.085 (2)	0.609 (4)
H87C	−0.066081	0.508980	0.552718	0.102*	0.609 (4)
H87D	−0.173175	0.501398	0.518011	0.102*	0.609 (4)
C88'	−0.1352 (6)	0.54173 (19)	0.4404 (5)	0.0597 (19)	0.609 (4)
H88C	−0.101999	0.531100	0.398034	0.072*	0.609 (4)
H88D	−0.199375	0.544161	0.403442	0.072*	0.609 (4)

### Atomic displacement parameters (Å^2^)

**Table:**

	*U*^11^	*U*^22^	*U*^33^	*U*^12^	*U*^13^	*U*^23^
Co1	0.02125 (18)	0.01477 (17)	0.01464 (16)	0.00069 (13)	0.00911 (13)	0.00017 (12)
P1	0.0234 (3)	0.0164 (2)	0.0162 (2)	−0.00055 (19)	0.0087 (2)	−0.00049 (18)
P2	0.0241 (3)	0.0183 (3)	0.0165 (2)	0.0010 (2)	0.0104 (2)	−0.00129 (19)
P3	0.0245 (3)	0.0140 (2)	0.0164 (2)	0.00024 (19)	0.0102 (2)	0.00049 (18)
P4	0.0234 (3)	0.0152 (2)	0.0155 (2)	0.00125 (19)	0.0091 (2)	0.00117 (18)
C1	0.0258 (11)	0.0219 (11)	0.0202 (10)	0.0008 (8)	0.0100 (8)	0.0019 (8)
C2	0.0251 (12)	0.0293 (12)	0.0284 (11)	−0.0031 (9)	0.0101 (9)	−0.0021 (9)
C3	0.0230 (12)	0.0400 (14)	0.0349 (13)	−0.0034 (10)	0.0110 (10)	−0.0006 (11)
C4	0.0272 (13)	0.0466 (16)	0.0372 (14)	0.0029 (11)	0.0179 (11)	−0.0023 (12)
C5	0.0257 (12)	0.0358 (13)	0.0281 (12)	0.0011 (10)	0.0146 (10)	−0.0041 (10)
C6	0.0279 (12)	0.0232 (11)	0.0204 (10)	0.0005 (9)	0.0119 (9)	0.0012 (8)
C7	0.0246 (11)	0.0194 (10)	0.0181 (10)	0.0025 (8)	0.0096 (8)	0.0005 (8)
C8	0.0249 (11)	0.0205 (10)	0.0214 (10)	0.0024 (8)	0.0100 (9)	0.0014 (8)
C9	0.0295 (12)	0.0256 (11)	0.0266 (11)	0.0038 (9)	0.0130 (9)	0.0066 (9)
C10	0.0294 (12)	0.0338 (13)	0.0178 (10)	0.0068 (10)	0.0094 (9)	0.0004 (9)
C11	0.0278 (12)	0.0237 (11)	0.0227 (11)	0.0044 (9)	0.0069 (9)	−0.0028 (9)
C12	0.0270 (11)	0.0201 (10)	0.0217 (10)	0.0028 (9)	0.0081 (9)	0.0010 (8)
C13	0.0290 (12)	0.0212 (11)	0.0166 (9)	−0.0013 (9)	0.0063 (8)	0.0023 (8)
C14	0.0335 (12)	0.0193 (11)	0.0208 (10)	−0.0002 (9)	0.0074 (9)	0.0008 (8)
C15	0.0421 (14)	0.0213 (11)	0.0246 (11)	0.0037 (10)	0.0023 (10)	−0.0011 (9)
C16	0.0441 (15)	0.0173 (11)	0.0352 (13)	−0.0042 (10)	0.0004 (11)	0.0071 (10)
C17	0.0374 (14)	0.0322 (13)	0.0319 (13)	−0.0078 (11)	0.0057 (11)	0.0132 (11)
C18	0.0326 (13)	0.0266 (12)	0.0251 (11)	−0.0028 (10)	0.0099 (10)	0.0059 (9)
C19	0.0223 (11)	0.0269 (11)	0.0213 (10)	−0.0016 (9)	0.0101 (8)	−0.0001 (9)
C20	0.0381 (14)	0.0301 (12)	0.0215 (11)	−0.0004 (10)	0.0175 (10)	−0.0015 (9)
C21	0.0447 (15)	0.0408 (15)	0.0233 (12)	−0.0049 (12)	0.0178 (11)	−0.0050 (10)
C22	0.0359 (14)	0.0452 (15)	0.0210 (11)	−0.0046 (11)	0.0131 (10)	0.0061 (10)
C23	0.0299 (13)	0.0325 (13)	0.0269 (12)	−0.0017 (10)	0.0113 (10)	0.0070 (10)
C24	0.0252 (11)	0.0275 (12)	0.0227 (11)	−0.0021 (9)	0.0126 (9)	0.0003 (9)
C25	0.0305 (12)	0.0212 (11)	0.0198 (10)	0.0015 (9)	0.0144 (9)	−0.0011 (8)
C26	0.0336 (13)	0.0265 (12)	0.0323 (12)	0.0050 (10)	0.0110 (10)	−0.0008 (10)
C27	0.0443 (16)	0.0272 (13)	0.0470 (15)	0.0134 (11)	0.0159 (13)	0.0037 (11)
C28	0.0541 (17)	0.0200 (12)	0.0433 (15)	0.0052 (11)	0.0251 (13)	−0.0024 (10)
C29	0.0461 (15)	0.0240 (12)	0.0292 (12)	−0.0046 (10)	0.0166 (11)	−0.0058 (10)
C30	0.0377 (14)	0.0226 (11)	0.0237 (11)	0.0007 (10)	0.0117 (10)	−0.0012 (9)
C31	0.0289 (11)	0.0172 (10)	0.0173 (9)	0.0004 (8)	0.0115 (8)	−0.0022 (8)
C32	0.0253 (11)	0.0219 (11)	0.0258 (11)	−0.0009 (9)	0.0132 (9)	0.0004 (9)
C33	0.0271 (12)	0.0269 (12)	0.0320 (12)	−0.0045 (9)	0.0155 (10)	−0.0031 (9)
C34	0.0238 (12)	0.0276 (12)	0.0289 (12)	0.0032 (9)	0.0082 (9)	−0.0016 (9)
C35	0.0252 (11)	0.0221 (11)	0.0229 (10)	0.0018 (9)	0.0090 (9)	−0.0005 (8)
C36	0.0277 (11)	0.0184 (10)	0.0167 (9)	−0.0004 (8)	0.0107 (8)	−0.0023 (8)
C37	0.0290 (11)	0.0189 (10)	0.0167 (9)	0.0006 (8)	0.0129 (8)	−0.0012 (8)
C38	0.0328 (13)	0.0219 (11)	0.0225 (10)	0.0034 (9)	0.0084 (9)	−0.0031 (9)
C39	0.0413 (14)	0.0242 (12)	0.0225 (11)	0.0094 (10)	0.0107 (10)	0.0002 (9)
C40	0.0489 (16)	0.0163 (11)	0.0331 (13)	0.0034 (10)	0.0185 (11)	−0.0026 (9)
C41	0.0405 (15)	0.0237 (13)	0.0464 (15)	−0.0011 (11)	0.0099 (12)	−0.0118 (11)
C42	0.0354 (14)	0.0219 (12)	0.0319 (12)	0.0011 (10)	0.0038 (10)	−0.0070 (10)
C43	0.0248 (11)	0.0163 (10)	0.0212 (10)	−0.0001 (8)	0.0109 (8)	−0.0001 (8)
C44	0.0359 (13)	0.0195 (10)	0.0209 (10)	−0.0001 (9)	0.0162 (9)	0.0014 (8)
C45	0.0427 (14)	0.0253 (12)	0.0245 (11)	0.0028 (10)	0.0164 (10)	0.0050 (9)
C46	0.0342 (13)	0.0316 (13)	0.0189 (10)	0.0031 (10)	0.0136 (9)	−0.0015 (9)
C47	0.0305 (12)	0.0212 (11)	0.0256 (11)	0.0013 (9)	0.0123 (9)	−0.0061 (9)
C48	0.0302 (12)	0.0172 (10)	0.0216 (10)	0.0009 (8)	0.0113 (9)	0.0012 (8)
C49	0.0270 (11)	0.0177 (10)	0.0176 (10)	0.0006 (8)	0.0064 (8)	0.0003 (8)
C50	0.0310 (12)	0.0195 (10)	0.0221 (10)	−0.0001 (9)	0.0144 (9)	−0.0001 (8)
C51	0.0356 (13)	0.0229 (11)	0.0296 (12)	0.0013 (10)	0.0166 (10)	−0.0036 (9)
C52	0.0355 (13)	0.0161 (10)	0.0295 (12)	0.0021 (9)	0.0096 (10)	−0.0024 (9)
C53	0.0313 (12)	0.0178 (11)	0.0269 (11)	−0.0012 (9)	0.0077 (9)	0.0044 (9)
C54	0.0256 (11)	0.0203 (11)	0.0213 (10)	0.0004 (8)	0.0091 (8)	0.0015 (8)
C55	0.0238 (11)	0.0231 (11)	0.0180 (10)	−0.0003 (8)	0.0103 (8)	0.0004 (8)
C56	0.0332 (12)	0.0233 (11)	0.0208 (10)	0.0035 (9)	0.0100 (9)	0.0015 (8)
C57	0.0360 (13)	0.0271 (12)	0.0232 (11)	0.0002 (10)	0.0083 (10)	0.0049 (9)
C58	0.0341 (13)	0.0364 (13)	0.0155 (10)	−0.0030 (10)	0.0092 (9)	0.0014 (9)
C59	0.0279 (12)	0.0273 (12)	0.0238 (11)	−0.0022 (9)	0.0118 (9)	−0.0031 (9)
C60	0.0244 (11)	0.0215 (11)	0.0215 (10)	0.0002 (8)	0.0094 (9)	0.0015 (8)
Br1	0.04265 (19)	0.02299 (15)	0.1025 (3)	−0.00182 (12)	0.00695 (18)	0.01037 (16)
Mg1	0.0286 (4)	0.0227 (4)	0.0349 (4)	−0.0027 (3)	0.0092 (3)	−0.0004 (3)
O1	0.0312 (9)	0.0215 (8)	0.0269 (8)	−0.0027 (7)	0.0102 (7)	−0.0027 (6)
O2	0.0312 (18)	0.0277 (11)	0.039 (3)	−0.0074 (14)	0.0084 (18)	−0.005 (2)
C65	0.0319 (17)	0.028 (2)	0.070 (6)	−0.0095 (15)	0.013 (4)	0.000 (4)
C66	0.055 (3)	0.030 (4)	0.086 (7)	−0.014 (3)	−0.001 (4)	0.002 (4)
C67	0.063 (4)	0.023 (2)	0.049 (2)	0.011 (2)	−0.022 (3)	−0.0091 (18)
C68	0.047 (5)	0.0291 (19)	0.037 (3)	0.004 (2)	−0.003 (2)	−0.0057 (17)
O2'	0.0312 (18)	0.0277 (11)	0.039 (3)	−0.0074 (14)	0.0084 (18)	−0.005 (2)
C65'	0.0319 (17)	0.028 (2)	0.070 (6)	−0.0095 (15)	0.013 (4)	0.000 (4)
C66'	0.055 (3)	0.030 (4)	0.086 (7)	−0.014 (3)	−0.001 (4)	0.002 (4)
C67'	0.063 (4)	0.023 (2)	0.049 (2)	0.011 (2)	−0.022 (3)	−0.0091 (18)
C68'	0.047 (5)	0.0291 (19)	0.037 (3)	0.004 (2)	−0.003 (2)	−0.0057 (17)
O3	0.034 (5)	0.035 (2)	0.030 (3)	0.004 (4)	0.006 (4)	−0.013 (3)
C69	0.0457 (19)	0.060 (2)	0.047 (5)	−0.0039 (16)	0.027 (3)	−0.003 (3)
C70	0.065 (4)	0.120 (5)	0.051 (4)	0.035 (4)	0.039 (3)	0.015 (4)
C71	0.113 (9)	0.124 (6)	0.044 (5)	0.071 (5)	0.028 (5)	−0.007 (4)
C72	0.088 (7)	0.060 (2)	0.051 (3)	0.026 (3)	0.007 (5)	−0.032 (2)
O3'	0.034 (5)	0.035 (2)	0.030 (3)	0.004 (4)	0.006 (4)	−0.013 (3)
C69'	0.0457 (19)	0.060 (2)	0.047 (5)	−0.0039 (16)	0.027 (3)	−0.003 (3)
C70'	0.065 (4)	0.120 (5)	0.051 (4)	0.035 (4)	0.039 (3)	0.015 (4)
C71'	0.113 (9)	0.124 (6)	0.044 (5)	0.071 (5)	0.028 (5)	−0.007 (4)
C72'	0.088 (7)	0.060 (2)	0.051 (3)	0.026 (3)	0.007 (5)	−0.032 (2)
O4	0.0291 (9)	0.0343 (10)	0.0292 (9)	0.0012 (7)	0.0082 (7)	−0.0039 (7)
O5	0.0358 (10)	0.0463 (11)	0.0411 (10)	0.0019 (8)	0.0197 (8)	0.0146 (9)
C61	0.0407 (15)	0.0279 (13)	0.0362 (13)	−0.0042 (11)	0.0068 (11)	−0.0085 (11)
C62	0.0438 (16)	0.0248 (13)	0.0461 (15)	0.0012 (11)	0.0096 (12)	−0.0080 (11)
C63	0.0471 (16)	0.0230 (12)	0.0421 (15)	−0.0025 (11)	0.0107 (12)	0.0026 (11)
C64	0.0365 (14)	0.0242 (12)	0.0300 (12)	−0.0012 (10)	0.0069 (10)	0.0006 (9)
C73	0.0326 (14)	0.0311 (14)	0.0503 (16)	0.0035 (11)	0.0079 (12)	−0.0040 (12)
C74	0.080 (3)	0.0407 (18)	0.055 (2)	0.0168 (18)	−0.0041 (18)	−0.0001 (15)
C75	0.0474 (17)	0.0438 (16)	0.0341 (14)	−0.0034 (13)	0.0050 (12)	0.0079 (12)
C76	0.0408 (15)	0.0336 (14)	0.0273 (12)	−0.0023 (11)	0.0088 (11)	−0.0018 (10)
C77	0.051 (4)	0.064 (3)	0.056 (2)	−0.006 (2)	0.023 (2)	0.027 (2)
C78	0.051 (6)	0.109 (5)	0.039 (4)	0.014 (4)	0.022 (4)	0.022 (3)
C77'	0.051 (4)	0.064 (3)	0.056 (2)	−0.006 (2)	0.023 (2)	0.027 (2)
C78'	0.051 (6)	0.109 (5)	0.039 (4)	0.014 (4)	0.022 (4)	0.022 (3)
C79	0.0411 (18)	0.086 (3)	0.0442 (18)	0.0067 (17)	0.0147 (14)	−0.0014 (17)
C80	0.0328 (15)	0.060 (2)	0.0445 (16)	0.0087 (13)	0.0185 (12)	0.0094 (14)
O6	0.0610 (14)	0.0383 (11)	0.0480 (12)	−0.0062 (10)	0.0235 (10)	−0.0034 (9)
C81	0.056 (2)	0.063 (2)	0.0456 (17)	−0.0177 (17)	0.0202 (15)	−0.0021 (16)
C82	0.0487 (18)	0.0391 (16)	0.0546 (19)	0.0049 (14)	0.0058 (15)	−0.0066 (14)
C83	0.0475 (19)	0.0470 (19)	0.096 (3)	−0.0077 (15)	0.0401 (19)	−0.0279 (19)
C84	0.0416 (18)	0.061 (2)	0.067 (2)	−0.0089 (15)	0.0075 (16)	0.0274 (18)
O7	0.071 (3)	0.059 (2)	0.058 (3)	−0.005 (2)	0.040 (3)	0.004 (2)
C85	0.043 (2)	0.065 (5)	0.042 (3)	0.007 (3)	0.013 (2)	−0.003 (3)
C86	0.044 (4)	0.086 (7)	0.051 (4)	0.006 (3)	0.018 (3)	0.027 (4)
C87	0.139 (8)	0.056 (3)	0.075 (5)	0.017 (4)	0.054 (5)	0.025 (4)
C88	0.083 (6)	0.059 (3)	0.040 (3)	0.027 (4)	0.021 (4)	0.009 (2)
O7'	0.071 (3)	0.059 (2)	0.058 (3)	−0.005 (2)	0.040 (3)	0.004 (2)
C85'	0.043 (2)	0.065 (5)	0.042 (3)	0.007 (3)	0.013 (2)	−0.003 (3)
C86'	0.044 (4)	0.086 (7)	0.051 (4)	0.006 (3)	0.018 (3)	0.027 (4)
C87'	0.139 (8)	0.056 (3)	0.075 (5)	0.017 (4)	0.054 (5)	0.025 (4)
C88'	0.083 (6)	0.059 (3)	0.040 (3)	0.027 (4)	0.021 (4)	0.009 (2)

### Geometric parameters (Å, º)

**Table:**

Co1—P1	2.1049 (6)	C65—H65A	0.9700
Co1—P2	2.0988 (6)	C65—H65B	0.9700
Co1—P3	2.0968 (6)	C65—C66	1.516 (8)
Co1—P4	2.1050 (6)	C66—H66A	0.9700
P1—C1	1.870 (2)	C66—H66B	0.9700
P1—C7	1.876 (2)	C66—C67	1.532 (10)
P1—C13	1.849 (2)	C67—H67A	0.9700
P2—C6	1.854 (2)	C67—H67B	0.9700
P2—C19	1.867 (2)	C67—C68	1.517 (7)
P2—C25	1.847 (2)	C68—H68A	0.9700
P3—C31	1.859 (2)	C68—H68B	0.9700
P3—C37	1.846 (2)	O2'—C65'	1.456 (13)
P3—C43	1.870 (2)	O2'—C68'	1.441 (12)
P4—C36	1.863 (2)	C65'—H65C	0.9700
P4—C49	1.843 (2)	C65'—H65D	0.9700
P4—C55	1.874 (2)	C65'—C66'	1.523 (14)
C1—C2	1.399 (3)	C66'—H66C	0.9700
C1—C6	1.396 (3)	C66'—H66D	0.9700
C2—H2	0.9300	C66'—C67'	1.518 (14)
C2—C3	1.386 (4)	C67'—H67C	0.9700
C3—H3	0.9300	C67'—H67D	0.9700
C3—C4	1.391 (4)	C67'—C68'	1.512 (12)
C4—H4	0.9300	C68'—H68C	0.9700
C4—C5	1.380 (4)	C68'—H68D	0.9700
C5—H5	0.9300	O3—C69	1.458 (10)
C5—C6	1.402 (3)	O3—C72	1.466 (8)
C7—C8	1.397 (3)	C69—H69A	0.9700
C7—C12	1.396 (3)	C69—H69B	0.9700
C8—H8	0.9300	C69—C70	1.500 (14)
C8—C9	1.392 (3)	C70—H70A	0.9700
C9—H9	0.9300	C70—H70B	0.9700
C9—C10	1.388 (4)	C70—C71	1.494 (11)
C10—H10	0.9300	C71—H71A	0.9700
C10—C11	1.391 (3)	C71—H71B	0.9700
C11—H11	0.9300	C71—C72	1.491 (13)
C11—C12	1.393 (3)	C72—H72A	0.9700
C12—H12	0.9300	C72—H72B	0.9700
C13—C14	1.396 (3)	O3'—C69'	1.458 (13)
C13—C18	1.398 (3)	O3'—C72'	1.465 (13)
C14—H14	0.9300	C69'—H69C	0.9700
C14—C15	1.388 (3)	C69'—H69D	0.9700
C15—H15	0.9300	C69'—C70'	1.502 (17)
C15—C16	1.390 (4)	C70'—H70C	0.9700
C16—H16	0.9300	C70'—H70D	0.9700
C16—C17	1.378 (4)	C70'—C71'	1.500 (16)
C17—H17	0.9300	C71'—H71C	0.9700
C17—C18	1.391 (4)	C71'—H71D	0.9700
C18—H18	0.9300	C71'—C72'	1.500 (17)
C19—C20	1.396 (3)	C72'—H72C	0.9700
C19—C24	1.397 (3)	C72'—H72D	0.9700
C20—H20	0.9300	O4—C73	1.457 (3)
C20—C21	1.395 (3)	O4—C76	1.450 (3)
C21—H21	0.9300	O5—C77	1.449 (5)
C21—C22	1.386 (4)	O5—C77'	1.450 (8)
C22—H22	0.9300	O5—C80	1.450 (4)
C22—C23	1.386 (4)	C61—H61A	0.9700
C23—H23	0.9300	C61—H61B	0.9700
C23—C24	1.390 (3)	C61—C62	1.510 (4)
C24—H24	0.9300	C62—H62A	0.9700
C25—C26	1.394 (3)	C62—H62B	0.9700
C25—C30	1.396 (3)	C62—C63	1.511 (4)
C26—H26	0.9300	C63—H63A	0.9700
C26—C27	1.389 (4)	C63—H63B	0.9700
C27—H27	0.9300	C63—C64	1.512 (3)
C27—C28	1.377 (4)	C64—H64A	0.9700
C28—H28	0.9300	C64—H64B	0.9700
C28—C29	1.376 (4)	C73—H73A	0.9700
C29—H29	0.9300	C73—H73B	0.9700
C29—C30	1.389 (3)	C73—C74	1.495 (5)
C30—H30	0.9300	C74—H74A	0.9700
C31—C32	1.396 (3)	C74—H74B	0.9700
C31—C36	1.397 (3)	C74—C75	1.515 (5)
C32—H32	0.9300	C75—H75A	0.9700
C32—C33	1.387 (3)	C75—H75B	0.9700
C33—H33	0.9300	C75—C76	1.518 (4)
C33—C34	1.397 (3)	C76—H76A	0.9700
C34—H34	0.9300	C76—H76B	0.9700
C34—C35	1.386 (3)	C77—H77A	0.9700
C35—H35	0.9300	C77—H77B	0.9700
C35—C36	1.398 (3)	C77—C78	1.533 (8)
C37—C38	1.394 (3)	C78—H78A	0.9700
C37—C42	1.393 (3)	C78—H78B	0.9700
C38—H38	0.9300	C78—C79	1.525 (6)
C38—C39	1.386 (3)	C77'—H77C	0.9700
C39—H39	0.9300	C77'—H77D	0.9700
C39—C40	1.384 (4)	C77'—C78'	1.531 (9)
C40—H40	0.9300	C78'—H78C	0.9700
C40—C41	1.384 (4)	C78'—H78D	0.9700
C41—H41	0.9300	C78'—C79	1.539 (8)
C41—C42	1.387 (3)	C79—H79A	0.9700
C42—H42	0.9300	C79—H79B	0.9700
C43—C44	1.391 (3)	C79—H79C	0.9700
C43—C48	1.402 (3)	C79—H79D	0.9700
C44—H44	0.9300	C79—C80	1.504 (5)
C44—C45	1.391 (3)	C80—H80A	0.9700
C45—H45	0.9300	C80—H80B	0.9700
C45—C46	1.384 (3)	O6—C81	1.423 (4)
C46—H46	0.9300	O6—C84	1.423 (4)
C46—C47	1.388 (3)	C81—H81A	0.9700
C47—H47	0.9300	C81—H81B	0.9700
C47—C48	1.383 (3)	C81—C82	1.500 (5)
C48—H48	0.9300	C82—H82A	0.9700
C49—C50	1.399 (3)	C82—H82B	0.9700
C49—C54	1.404 (3)	C82—C83	1.511 (5)
C50—H50	0.9300	C83—H83A	0.9700
C50—C51	1.387 (3)	C83—H83B	0.9700
C51—H51	0.9300	C83—C84	1.510 (6)
C51—C52	1.386 (3)	C84—H84A	0.9700
C52—H52	0.9300	C84—H84B	0.9700
C52—C53	1.386 (3)	O7—C85	1.422 (10)
C53—H53	0.9300	O7—C88	1.409 (11)
C53—C54	1.388 (3)	C85—H85A	0.9700
C54—H54	0.9300	C85—H85B	0.9700
C55—C56	1.393 (3)	C85—C86	1.508 (12)
C55—C60	1.399 (3)	C86—H86A	0.9700
C56—H56	0.9300	C86—H86B	0.9700
C56—C57	1.391 (3)	C86—C87	1.506 (13)
C57—H57	0.9300	C87—H87A	0.9700
C57—C58	1.383 (4)	C87—H87B	0.9700
C58—H58	0.9300	C87—C88	1.492 (13)
C58—C59	1.390 (3)	C88—H88A	0.9700
C59—H59	0.9300	C88—H88B	0.9700
C59—C60	1.383 (3)	O7'—C85'	1.417 (7)
C60—H60	0.9300	O7'—C88'	1.372 (8)
Br1—Mg1	2.5990 (9)	C85'—H85C	0.9700
Mg1—O1	2.1166 (18)	C85'—H85D	0.9700
Mg1—O2	2.128 (6)	C85'—C86'	1.527 (8)
Mg1—O2'	2.129 (12)	C86'—H86C	0.9700
Mg1—O3	2.132 (7)	C86'—H86D	0.9700
Mg1—O3'	2.114 (11)	C86'—C87'	1.507 (11)
Mg1—O4	2.1177 (19)	C87'—H87C	0.9700
Mg1—O5	2.126 (2)	C87'—H87D	0.9700
O1—C61	1.445 (3)	C87'—C88'	1.500 (8)
O1—C64	1.460 (3)	C88'—H88C	0.9700
O2—C65	1.464 (7)	C88'—H88D	0.9700
O2—C68	1.450 (7)		
			
P1—Co1—P4	120.60 (3)	O2—C68—H68A	111.3
P2—Co1—P1	90.18 (2)	O2—C68—H68B	111.3
P2—Co1—P4	118.59 (2)	C67—C68—H68A	111.3
P3—Co1—P1	122.08 (3)	C67—C68—H68B	111.3
P3—Co1—P2	118.74 (3)	H68A—C68—H68B	109.2
P3—Co1—P4	89.75 (2)	C65'—O2'—Mg1	120 (2)
C1—P1—Co1	109.40 (8)	C68'—O2'—Mg1	128.5 (14)
C1—P1—C7	98.00 (10)	C68'—O2'—C65'	106.5 (12)
C7—P1—Co1	126.18 (7)	O2'—C65'—H65C	110.9
C13—P1—Co1	121.21 (8)	O2'—C65'—H65D	110.9
C13—P1—C1	100.06 (10)	O2'—C65'—C66'	104.3 (14)
C13—P1—C7	97.06 (10)	H65C—C65'—H65D	108.9
C6—P2—Co1	109.95 (7)	C66'—C65'—H65C	110.9
C6—P2—C19	97.01 (10)	C66'—C65'—H65D	110.9
C19—P2—Co1	121.38 (8)	C65'—C66'—H66C	111.4
C25—P2—Co1	122.03 (7)	C65'—C66'—H66D	111.4
C25—P2—C6	101.73 (11)	H66C—C66'—H66D	109.2
C25—P2—C19	100.47 (10)	C67'—C66'—C65'	102.0 (11)
C31—P3—Co1	110.35 (7)	C67'—C66'—H66C	111.4
C31—P3—C43	95.75 (10)	C67'—C66'—H66D	111.4
C37—P3—Co1	121.35 (7)	C66'—C67'—H67C	110.2
C37—P3—C31	103.36 (10)	C66'—C67'—H67D	110.2
C37—P3—C43	98.79 (9)	H67C—C67'—H67D	108.5
C43—P3—Co1	122.95 (7)	C68'—C67'—C66'	107.3 (9)
C36—P4—Co1	109.46 (7)	C68'—C67'—H67C	110.2
C36—P4—C55	96.99 (10)	C68'—C67'—H67D	110.2
C49—P4—Co1	121.52 (7)	O2'—C68'—C67'	105.3 (11)
C49—P4—C36	100.76 (10)	O2'—C68'—H68C	110.7
C49—P4—C55	99.23 (10)	O2'—C68'—H68D	110.7
C55—P4—Co1	124.14 (7)	C67'—C68'—H68C	110.7
C2—C1—P1	125.93 (18)	C67'—C68'—H68D	110.7
C6—C1—P1	114.93 (17)	H68C—C68'—H68D	108.8
C6—C1—C2	119.1 (2)	C69—O3—Mg1	124.7 (11)
C1—C2—H2	119.7	C69—O3—C72	107.3 (7)
C3—C2—C1	120.6 (2)	C72—O3—Mg1	128.1 (13)
C3—C2—H2	119.7	O3—C69—H69A	110.5
C2—C3—H3	120.0	O3—C69—H69B	110.5
C2—C3—C4	120.0 (2)	O3—C69—C70	106.3 (8)
C4—C3—H3	120.0	H69A—C69—H69B	108.7
C3—C4—H4	120.0	C70—C69—H69A	110.5
C5—C4—C3	120.0 (2)	C70—C69—H69B	110.5
C5—C4—H4	120.0	C69—C70—H70A	111.2
C4—C5—H5	119.8	C69—C70—H70B	111.2
C4—C5—C6	120.4 (2)	H70A—C70—H70B	109.1
C6—C5—H5	119.8	C71—C70—C69	102.9 (10)
C1—C6—P2	115.32 (17)	C71—C70—H70A	111.2
C1—C6—C5	119.8 (2)	C71—C70—H70B	111.2
C5—C6—P2	124.85 (18)	C70—C71—H71A	111.1
C8—C7—P1	117.54 (17)	C70—C71—H71B	111.1
C12—C7—P1	124.33 (16)	H71A—C71—H71B	109.0
C12—C7—C8	118.1 (2)	C72—C71—C70	103.5 (12)
C7—C8—H8	119.6	C72—C71—H71A	111.1
C9—C8—C7	120.7 (2)	C72—C71—H71B	111.1
C9—C8—H8	119.6	O3—C72—C71	106.9 (8)
C8—C9—H9	119.7	O3—C72—H72A	110.3
C10—C9—C8	120.7 (2)	O3—C72—H72B	110.3
C10—C9—H9	119.7	C71—C72—H72A	110.3
C9—C10—H10	120.4	C71—C72—H72B	110.3
C9—C10—C11	119.2 (2)	H72A—C72—H72B	108.6
C11—C10—H10	120.4	C69'—O3'—Mg1	119.6 (19)
C10—C11—H11	119.9	C69'—O3'—C72'	108.5 (13)
C10—C11—C12	120.1 (2)	C72'—O3'—Mg1	126 (2)
C12—C11—H11	119.9	O3'—C69'—H69C	110.7
C7—C12—H12	119.4	O3'—C69'—H69D	110.7
C11—C12—C7	121.2 (2)	O3'—C69'—C70'	105.3 (11)
C11—C12—H12	119.4	H69C—C69'—H69D	108.8
C14—C13—P1	118.76 (17)	C70'—C69'—H69C	110.7
C14—C13—C18	118.1 (2)	C70'—C69'—H69D	110.7
C18—C13—P1	123.06 (18)	C69'—C70'—H70C	111.4
C13—C14—H14	119.5	C69'—C70'—H70D	111.4
C15—C14—C13	121.1 (2)	H70C—C70'—H70D	109.3
C15—C14—H14	119.5	C71'—C70'—C69'	101.9 (15)
C14—C15—H15	119.9	C71'—C70'—H70C	111.4
C14—C15—C16	120.2 (2)	C71'—C70'—H70D	111.4
C16—C15—H15	119.9	C70'—C71'—H71C	111.5
C15—C16—H16	120.4	C70'—C71'—H71D	111.5
C17—C16—C15	119.1 (2)	C70'—C71'—C72'	101 (2)
C17—C16—H16	120.4	H71C—C71'—H71D	109.3
C16—C17—H17	119.5	C72'—C71'—H71C	111.5
C16—C17—C18	121.0 (2)	C72'—C71'—H71D	111.5
C18—C17—H17	119.5	O3'—C72'—C71'	102.3 (14)
C13—C18—H18	119.8	O3'—C72'—H72C	111.3
C17—C18—C13	120.4 (2)	O3'—C72'—H72D	111.3
C17—C18—H18	119.8	C71'—C72'—H72C	111.3
C20—C19—P2	125.82 (18)	C71'—C72'—H72D	111.3
C20—C19—C24	118.3 (2)	H72C—C72'—H72D	109.2
C24—C19—P2	115.87 (16)	C73—O4—Mg1	119.83 (16)
C19—C20—H20	119.8	C76—O4—Mg1	132.79 (16)
C21—C20—C19	120.5 (2)	C76—O4—C73	106.4 (2)
C21—C20—H20	119.8	C77—O5—Mg1	124.1 (6)
C20—C21—H21	119.7	C77—O5—C80	105.3 (7)
C22—C21—C20	120.5 (2)	C77'—O5—Mg1	123.2 (9)
C22—C21—H21	119.7	C77'—O5—C80	106.9 (10)
C21—C22—H22	120.3	C80—O5—Mg1	129.57 (16)
C23—C22—C21	119.5 (2)	O1—C61—H61A	110.6
C23—C22—H22	120.3	O1—C61—H61B	110.6
C22—C23—H23	119.9	O1—C61—C62	105.8 (2)
C22—C23—C24	120.2 (2)	H61A—C61—H61B	108.7
C24—C23—H23	119.9	C62—C61—H61A	110.6
C19—C24—H24	119.5	C62—C61—H61B	110.6
C23—C24—C19	121.0 (2)	C61—C62—H62A	111.3
C23—C24—H24	119.5	C61—C62—H62B	111.3
C26—C25—P2	124.51 (19)	C61—C62—C63	102.3 (2)
C26—C25—C30	117.5 (2)	H62A—C62—H62B	109.2
C30—C25—P2	117.65 (18)	C63—C62—H62A	111.3
C25—C26—H26	119.6	C63—C62—H62B	111.3
C27—C26—C25	120.9 (3)	C62—C63—H63A	111.4
C27—C26—H26	119.6	C62—C63—H63B	111.4
C26—C27—H27	119.6	C62—C63—C64	102.0 (2)
C28—C27—C26	120.8 (3)	H63A—C63—H63B	109.2
C28—C27—H27	119.6	C64—C63—H63A	111.4
C27—C28—H28	120.4	C64—C63—H63B	111.4
C29—C28—C27	119.2 (2)	O1—C64—C63	105.8 (2)
C29—C28—H28	120.4	O1—C64—H64A	110.6
C28—C29—H29	119.8	O1—C64—H64B	110.6
C28—C29—C30	120.4 (3)	C63—C64—H64A	110.6
C30—C29—H29	119.8	C63—C64—H64B	110.6
C25—C30—H30	119.4	H64A—C64—H64B	108.7
C29—C30—C25	121.2 (2)	O4—C73—H73A	110.3
C29—C30—H30	119.4	O4—C73—H73B	110.3
C32—C31—P3	125.87 (17)	O4—C73—C74	107.1 (3)
C32—C31—C36	119.7 (2)	H73A—C73—H73B	108.5
C36—C31—P3	114.28 (17)	C74—C73—H73A	110.3
C31—C32—H32	119.6	C74—C73—H73B	110.3
C33—C32—C31	120.8 (2)	C73—C74—H74A	110.5
C33—C32—H32	119.6	C73—C74—H74B	110.5
C32—C33—H33	120.2	C73—C74—C75	106.2 (3)
C32—C33—C34	119.5 (2)	H74A—C74—H74B	108.7
C34—C33—H33	120.2	C75—C74—H74A	110.5
C33—C34—H34	120.1	C75—C74—H74B	110.5
C35—C34—C33	119.8 (2)	C74—C75—H75A	111.1
C35—C34—H34	120.1	C74—C75—H75B	111.1
C34—C35—H35	119.5	C74—C75—C76	103.5 (2)
C34—C35—C36	121.0 (2)	H75A—C75—H75B	109.0
C36—C35—H35	119.5	C76—C75—H75A	111.1
C31—C36—P4	115.53 (17)	C76—C75—H75B	111.1
C31—C36—C35	119.1 (2)	O4—C76—C75	104.7 (2)
C35—C36—P4	125.39 (17)	O4—C76—H76A	110.8
C38—C37—P3	117.26 (17)	O4—C76—H76B	110.8
C42—C37—P3	125.07 (18)	C75—C76—H76A	110.8
C42—C37—C38	117.6 (2)	C75—C76—H76B	110.8
C37—C38—H38	119.2	H76A—C76—H76B	108.9
C39—C38—C37	121.5 (2)	O5—C77—H77A	110.8
C39—C38—H38	119.2	O5—C77—H77B	110.8
C38—C39—H39	120.0	O5—C77—C78	104.9 (8)
C40—C39—C38	120.1 (2)	H77A—C77—H77B	108.8
C40—C39—H39	120.0	C78—C77—H77A	110.8
C39—C40—H40	120.5	C78—C77—H77B	110.8
C39—C40—C41	119.1 (2)	C77—C78—H78A	110.9
C41—C40—H40	120.5	C77—C78—H78B	110.9
C40—C41—H41	119.7	H78A—C78—H78B	108.9
C40—C41—C42	120.6 (3)	C79—C78—C77	104.3 (7)
C42—C41—H41	119.7	C79—C78—H78A	110.9
C37—C42—H42	119.5	C79—C78—H78B	110.9
C41—C42—C37	121.0 (2)	O5—C77'—H77C	110.7
C41—C42—H42	119.5	O5—C77'—H77D	110.7
C44—C43—P3	125.10 (16)	O5—C77'—C78'	105.4 (14)
C44—C43—C48	117.9 (2)	H77C—C77'—H77D	108.8
C48—C43—P3	116.90 (16)	C78'—C77'—H77C	110.7
C43—C44—H44	119.5	C78'—C77'—H77D	110.7
C45—C44—C43	121.1 (2)	C77'—C78'—H78C	111.2
C45—C44—H44	119.5	C77'—C78'—H78D	111.2
C44—C45—H45	119.9	C77'—C78'—C79	103.0 (14)
C46—C45—C44	120.2 (2)	H78C—C78'—H78D	109.1
C46—C45—H45	119.9	C79—C78'—H78C	111.2
C45—C46—H46	120.3	C79—C78'—H78D	111.2
C45—C46—C47	119.3 (2)	C78—C79—H79A	110.7
C47—C46—H46	120.3	C78—C79—H79B	110.7
C46—C47—H47	119.7	C78'—C79—H79C	112.3
C48—C47—C46	120.5 (2)	C78'—C79—H79D	112.3
C48—C47—H47	119.7	H79A—C79—H79B	108.8
C43—C48—H48	119.6	H79C—C79—H79D	109.9
C47—C48—C43	120.8 (2)	C80—C79—C78	105.4 (5)
C47—C48—H48	119.6	C80—C79—C78'	97.5 (9)
C50—C49—P4	122.85 (17)	C80—C79—H79A	110.7
C50—C49—C54	117.6 (2)	C80—C79—H79B	110.7
C54—C49—P4	119.39 (17)	C80—C79—H79C	112.3
C49—C50—H50	119.5	C80—C79—H79D	112.3
C51—C50—C49	121.0 (2)	O5—C80—C79	104.3 (2)
C51—C50—H50	119.5	O5—C80—H80A	110.9
C50—C51—H51	119.6	O5—C80—H80B	110.9
C52—C51—C50	120.7 (2)	C79—C80—H80A	110.9
C52—C51—H51	119.6	C79—C80—H80B	110.9
C51—C52—H52	120.5	H80A—C80—H80B	108.9
C51—C52—C53	119.0 (2)	C81—O6—C84	105.6 (3)
C53—C52—H52	120.5	O6—C81—H81A	110.3
C52—C53—H53	119.7	O6—C81—H81B	110.3
C52—C53—C54	120.6 (2)	O6—C81—C82	106.9 (3)
C54—C53—H53	119.7	H81A—C81—H81B	108.6
C49—C54—H54	119.5	C82—C81—H81A	110.3
C53—C54—C49	120.9 (2)	C82—C81—H81B	110.3
C53—C54—H54	119.5	C81—C82—H82A	111.0
C56—C55—P4	126.10 (17)	C81—C82—H82B	111.0
C56—C55—C60	117.8 (2)	C81—C82—C83	103.9 (3)
C60—C55—P4	116.04 (16)	H82A—C82—H82B	109.0
C55—C56—H56	119.5	C83—C82—H82A	111.0
C57—C56—C55	121.0 (2)	C83—C82—H82B	111.0
C57—C56—H56	119.5	C82—C83—H83A	110.8
C56—C57—H57	119.8	C82—C83—H83B	110.8
C58—C57—C56	120.4 (2)	H83A—C83—H83B	108.8
C58—C57—H57	119.8	C84—C83—C82	104.9 (3)
C57—C58—H58	120.4	C84—C83—H83A	110.8
C57—C58—C59	119.2 (2)	C84—C83—H83B	110.8
C59—C58—H58	120.4	O6—C84—C83	107.3 (3)
C58—C59—H59	119.8	O6—C84—H84A	110.2
C60—C59—C58	120.4 (2)	O6—C84—H84B	110.2
C60—C59—H59	119.8	C83—C84—H84A	110.2
C55—C60—H60	119.4	C83—C84—H84B	110.2
C59—C60—C55	121.2 (2)	H84A—C84—H84B	108.5
C59—C60—H60	119.4	C88—O7—C85	108.9 (8)
O1—Mg1—Br1	175.61 (6)	O7—C85—H85A	110.5
O1—Mg1—O2	84.9 (4)	O7—C85—H85B	110.5
O1—Mg1—O2'	86.6 (8)	O7—C85—C86	106.0 (8)
O1—Mg1—O3	87.6 (6)	H85A—C85—H85B	108.7
O1—Mg1—O4	92.05 (8)	C86—C85—H85A	110.5
O1—Mg1—O5	85.15 (8)	C86—C85—H85B	110.5
O2—Mg1—Br1	90.8 (4)	C85—C86—H86A	111.1
O2—Mg1—O3	92.4 (5)	C85—C86—H86B	111.1
O2'—Mg1—Br1	89.0 (8)	H86A—C86—H86B	109.1
O3—Mg1—Br1	93.8 (6)	C87—C86—C85	103.4 (8)
O3'—Mg1—Br1	95.8 (11)	C87—C86—H86A	111.1
O3'—Mg1—O1	85.1 (11)	C87—C86—H86B	111.1
O3'—Mg1—O2'	93.5 (10)	C86—C87—H87A	111.5
O3'—Mg1—O4	92.3 (9)	C86—C87—H87B	111.5
O3'—Mg1—O5	170.2 (11)	H87A—C87—H87B	109.3
O4—Mg1—Br1	92.21 (6)	C88—C87—C86	101.4 (9)
O4—Mg1—O2	176.7 (4)	C88—C87—H87A	111.5
O4—Mg1—O2'	173.9 (6)	C88—C87—H87B	111.5
O4—Mg1—O3	86.2 (5)	O7—C88—C87	109.1 (8)
O4—Mg1—O5	87.55 (8)	O7—C88—H88A	109.9
O5—Mg1—Br1	93.96 (6)	O7—C88—H88B	109.9
O5—Mg1—O2	93.5 (3)	C87—C88—H88A	109.9
O5—Mg1—O2'	86.4 (6)	C87—C88—H88B	109.9
O5—Mg1—O3	170.2 (6)	H88A—C88—H88B	108.3
C61—O1—Mg1	126.68 (16)	C88'—O7'—C85'	107.3 (5)
C61—O1—C64	108.74 (19)	O7'—C85'—H85C	110.3
C64—O1—Mg1	124.51 (14)	O7'—C85'—H85D	110.3
C65—O2—Mg1	118.2 (9)	O7'—C85'—C86'	107.0 (5)
C68—O2—Mg1	127.7 (7)	H85C—C85'—H85D	108.6
C68—O2—C65	106.3 (6)	C86'—C85'—H85C	110.3
O2—C65—H65A	110.5	C86'—C85'—H85D	110.3
O2—C65—H65B	110.5	C85'—C86'—H86C	111.0
O2—C65—C66	106.3 (7)	C85'—C86'—H86D	111.0
H65A—C65—H65B	108.7	H86C—C86'—H86D	109.0
C66—C65—H65A	110.5	C87'—C86'—C85'	104.0 (5)
C66—C65—H65B	110.5	C87'—C86'—H86C	111.0
C65—C66—H66A	110.9	C87'—C86'—H86D	111.0
C65—C66—H66B	110.9	C86'—C87'—H87C	111.4
C65—C66—C67	104.2 (6)	C86'—C87'—H87D	111.4
H66A—C66—H66B	108.9	H87C—C87'—H87D	109.3
C67—C66—H66A	110.9	C88'—C87'—C86'	101.8 (5)
C67—C66—H66B	110.9	C88'—C87'—H87C	111.4
C66—C67—H67A	111.5	C88'—C87'—H87D	111.4
C66—C67—H67B	111.5	O7'—C88'—C87'	106.8 (6)
H67A—C67—H67B	109.3	O7'—C88'—H88C	110.4
C68—C67—C66	101.6 (5)	O7'—C88'—H88D	110.4
C68—C67—H67A	111.5	C87'—C88'—H88C	110.4
C68—C67—H67B	111.5	C87'—C88'—H88D	110.4
O2—C68—C67	102.2 (7)	H88C—C88'—H88D	108.6
			
Co1—P1—C1—C2	−176.18 (19)	C36—P4—C49—C54	−161.79 (18)
Co1—P1—C1—C6	3.97 (19)	C36—P4—C55—C56	102.6 (2)
Co1—P1—C7—C8	17.7 (2)	C36—P4—C55—C60	−79.98 (19)
Co1—P1—C7—C12	−162.17 (16)	C36—C31—C32—C33	2.7 (3)
Co1—P1—C13—C14	83.10 (19)	C37—P3—C31—C32	45.8 (2)
Co1—P1—C13—C18	−93.1 (2)	C37—P3—C31—C36	−138.16 (16)
Co1—P2—C6—C1	−2.31 (19)	C37—P3—C43—C44	−1.9 (2)
Co1—P2—C6—C5	178.27 (19)	C37—P3—C43—C48	175.40 (18)
Co1—P2—C19—C20	−142.05 (19)	C37—C38—C39—C40	2.6 (4)
Co1—P2—C19—C24	36.7 (2)	C38—C37—C42—C41	2.4 (4)
Co1—P2—C25—C26	−103.6 (2)	C38—C39—C40—C41	1.0 (4)
Co1—P2—C25—C30	69.39 (18)	C39—C40—C41—C42	−2.8 (4)
Co1—P3—C31—C32	176.95 (17)	C40—C41—C42—C37	1.1 (4)
Co1—P3—C31—C36	−6.98 (17)	C42—C37—C38—C39	−4.3 (3)
Co1—P3—C37—C38	66.63 (18)	C43—P3—C31—C32	−54.7 (2)
Co1—P3—C37—C42	−109.3 (2)	C43—P3—C31—C36	121.37 (16)
Co1—P3—C43—C44	−138.55 (19)	C43—P3—C37—C38	−70.98 (18)
Co1—P3—C43—C48	38.7 (2)	C43—P3—C37—C42	113.1 (2)
Co1—P4—C36—C31	3.70 (17)	C43—C44—C45—C46	2.6 (4)
Co1—P4—C36—C35	−176.30 (17)	C44—C43—C48—C47	1.6 (4)
Co1—P4—C49—C50	−98.33 (19)	C44—C45—C46—C47	0.6 (4)
Co1—P4—C49—C54	77.26 (19)	C45—C46—C47—C48	−2.6 (4)
Co1—P4—C55—C56	−138.13 (18)	C46—C47—C48—C43	1.5 (4)
Co1—P4—C55—C60	39.3 (2)	C48—C43—C44—C45	−3.6 (4)
P1—C1—C2—C3	179.60 (19)	C49—P4—C36—C31	−125.46 (16)
P1—C1—C6—P2	−1.1 (2)	C49—P4—C36—C35	54.5 (2)
P1—C1—C6—C5	178.40 (18)	C49—P4—C55—C56	0.5 (2)
P1—C7—C8—C9	−178.85 (17)	C49—P4—C55—C60	177.87 (18)
P1—C7—C12—C11	179.77 (17)	C49—C50—C51—C52	−0.1 (4)
P1—C13—C14—C15	−179.72 (18)	C50—C49—C54—C53	−4.1 (3)
P1—C13—C18—C17	178.61 (19)	C50—C51—C52—C53	−2.1 (4)
P2—C19—C20—C21	177.2 (2)	C51—C52—C53—C54	1.2 (4)
P2—C19—C24—C23	−178.42 (19)	C52—C53—C54—C49	2.0 (4)
P2—C25—C26—C27	174.1 (2)	C54—C49—C50—C51	3.1 (3)
P2—C25—C30—C29	−175.55 (18)	C55—P4—C36—C31	133.72 (17)
P3—C31—C32—C33	178.55 (17)	C55—P4—C36—C35	−46.3 (2)
P3—C31—C36—P4	2.0 (2)	C55—P4—C49—C50	121.6 (2)
P3—C31—C36—C35	−177.99 (16)	C55—P4—C49—C54	−62.79 (19)
P3—C37—C38—C39	179.49 (18)	C55—C56—C57—C58	1.1 (4)
P3—C37—C42—C41	178.3 (2)	C56—C55—C60—C59	1.6 (3)
P3—C43—C44—C45	173.6 (2)	C56—C57—C58—C59	0.4 (4)
P3—C43—C48—C47	−175.85 (19)	C57—C58—C59—C60	−0.9 (4)
P4—C49—C50—C51	178.80 (19)	C58—C59—C60—C55	−0.2 (4)
P4—C49—C54—C53	−179.91 (18)	C60—C55—C56—C57	−2.1 (4)
P4—C55—C56—C57	175.25 (19)	Mg1—O1—C61—C62	169.44 (17)
P4—C55—C60—C59	−175.99 (18)	Mg1—O1—C64—C63	165.75 (17)
C1—P1—C7—C8	−103.54 (18)	Mg1—O2—C65—C66	129.4 (10)
C1—P1—C7—C12	76.6 (2)	Mg1—O2—C68—C67	−107.0 (9)
C1—P1—C13—C14	−156.77 (19)	Mg1—O2'—C65'—C66'	117 (2)
C1—P1—C13—C18	27.0 (2)	Mg1—O2'—C68'—C67'	−124.5 (18)
C1—C2—C3—C4	1.3 (4)	Mg1—O3—C69—C70	165.0 (13)
C2—C1—C6—P2	179.09 (17)	Mg1—O3—C72—C71	172.0 (16)
C2—C1—C6—C5	−1.5 (3)	Mg1—O3'—C69'—C70'	−152 (2)
C2—C3—C4—C5	−0.1 (4)	Mg1—O3'—C72'—C71'	178 (2)
C3—C4—C5—C6	−1.9 (4)	Mg1—O4—C73—C74	145.5 (2)
C4—C5—C6—P2	−177.9 (2)	Mg1—O4—C76—C75	−133.0 (2)
C4—C5—C6—C1	2.7 (4)	Mg1—O5—C77—C78	−153.6 (9)
C6—P2—C19—C20	99.4 (2)	Mg1—O5—C77'—C78'	−178.6 (16)
C6—P2—C19—C24	−81.83 (19)	Mg1—O5—C80—C79	151.2 (2)
C6—P2—C25—C26	19.1 (2)	O1—C61—C62—C63	32.8 (3)
C6—P2—C25—C30	−167.86 (17)	O2—C65—C66—C67	−5.4 (15)
C6—C1—C2—C3	−0.6 (4)	C65—O2—C68—C67	41.1 (12)
C7—P1—C1—C2	−43.2 (2)	C65—C66—C67—C68	29.3 (10)
C7—P1—C1—C6	136.93 (17)	C66—C67—C68—O2	−43.0 (7)
C7—P1—C13—C14	−57.3 (2)	C68—O2—C65—C66	−22.3 (16)
C7—P1—C13—C18	126.4 (2)	O2'—C65'—C66'—C67'	33 (3)
C7—C8—C9—C10	−0.9 (4)	C65'—O2'—C68'—C67'	29 (3)
C8—C7—C12—C11	−0.1 (3)	C65'—C66'—C67'—C68'	−16 (2)
C8—C9—C10—C11	−0.2 (4)	C66'—C67'—C68'—O2'	−7 (2)
C9—C10—C11—C12	1.1 (3)	C68'—O2'—C65'—C66'	−39 (3)
C10—C11—C12—C7	−1.0 (3)	O3—C69—C70—C71	32 (2)
C12—C7—C8—C9	1.0 (3)	C69—O3—C72—C71	−8 (3)
C13—P1—C1—C2	55.5 (2)	C69—C70—C71—C72	−36 (3)
C13—P1—C1—C6	−124.38 (18)	C70—C71—C72—O3	28 (3)
C13—P1—C7—C8	155.21 (18)	C72—O3—C69—C70	−15 (3)
C13—P1—C7—C12	−24.6 (2)	O3'—C69'—C70'—C71'	−29 (4)
C13—C14—C15—C16	1.7 (4)	C69'—O3'—C72'—C71'	25 (5)
C14—C13—C18—C17	2.3 (4)	C69'—C70'—C71'—C72'	44 (4)
C14—C15—C16—C17	0.9 (4)	C70'—C71'—C72'—O3'	−42 (5)
C15—C16—C17—C18	−1.8 (4)	C72'—O3'—C69'—C70'	3 (5)
C16—C17—C18—C13	0.2 (4)	O4—C73—C74—C75	4.0 (4)
C18—C13—C14—C15	−3.3 (3)	O5—C77—C78—C79	−19.2 (19)
C19—P2—C6—C1	124.76 (18)	O5—C77'—C78'—C79	22 (3)
C19—P2—C6—C5	−54.7 (2)	C61—O1—C64—C63	−11.4 (3)
C19—P2—C25—C26	118.6 (2)	C61—C62—C63—C64	−38.8 (3)
C19—P2—C25—C30	−68.35 (19)	C62—C63—C64—O1	31.4 (3)
C19—C20—C21—C22	1.4 (4)	C64—O1—C61—C62	−13.5 (3)
C20—C19—C24—C23	0.4 (4)	C73—O4—C76—C75	35.2 (3)
C20—C21—C22—C23	−0.1 (4)	C73—C74—C75—C76	16.7 (4)
C21—C22—C23—C24	−1.0 (4)	C74—C75—C76—O4	−31.7 (3)
C22—C23—C24—C19	0.8 (4)	C76—O4—C73—C74	−24.5 (3)
C24—C19—C20—C21	−1.6 (4)	C77—O5—C80—C79	−40.3 (11)
C25—P2—C6—C1	−132.97 (17)	C77—C78—C79—C80	−4.8 (14)
C25—P2—C6—C5	47.6 (2)	C78—C79—C80—O5	27.1 (7)
C25—P2—C19—C20	−3.9 (2)	C77'—O5—C80—C79	−35 (2)
C25—P2—C19—C24	174.82 (18)	C77'—C78'—C79—C80	−41 (2)
C25—C26—C27—C28	0.5 (4)	C78'—C79—C80—O5	46.9 (8)
C26—C25—C30—C29	−2.0 (3)	C80—O5—C77—C78	37.2 (18)
C26—C27—C28—C29	−1.1 (4)	C80—O5—C77'—C78'	7 (3)
C27—C28—C29—C30	0.2 (4)	O6—C81—C82—C83	−25.5 (4)
C28—C29—C30—C25	1.4 (4)	C81—O6—C84—C83	−30.0 (4)
C30—C25—C26—C27	1.1 (4)	C81—C82—C83—C84	7.0 (4)
C31—P3—C37—C38	−169.09 (17)	C82—C83—C84—O6	13.6 (4)
C31—P3—C37—C42	15.0 (2)	C84—O6—C81—C82	34.9 (4)
C31—P3—C43—C44	102.6 (2)	O7—C85—C86—C87	−29.7 (12)
C31—P3—C43—C48	−80.09 (19)	C85—O7—C88—C87	7.5 (13)
C31—C32—C33—C34	−2.2 (3)	C85—C86—C87—C88	32.6 (12)
C32—C31—C36—P4	178.34 (16)	C86—C87—C88—O7	−25.5 (13)
C32—C31—C36—C35	−1.7 (3)	C88—O7—C85—C86	14.1 (12)
C32—C33—C34—C35	0.7 (4)	O7'—C85'—C86'—C87'	1.2 (8)
C33—C34—C35—C36	0.3 (3)	C85'—O7'—C88'—C87'	−36.0 (8)
C34—C35—C36—P4	−179.81 (17)	C85'—C86'—C87'—C88'	−20.8 (9)
C34—C35—C36—C31	0.2 (3)	C86'—C87'—C88'—O7'	35.3 (9)
C36—P4—C49—C50	22.6 (2)	C88'—O7'—C85'—C86'	21.4 (7)

### Hydrogen-bond geometry (Å, º)

**Table:**

*D*—H···*A*	*D*—H	H···*A*	*D*···*A*	*D*—H···*A*
C62—H62*B*···O6	0.97	2.48	3.438 (4)	167
C63—H63*B*···O7	0.97	2.59	3.555 (8)	179
C63—H63*B*···O7′	0.97	2.63	3.565 (6)	162
